# Machine Learning on Suicidal Risk and OCD: A Long-Term Follow-Up

**DOI:** 10.1192/j.eurpsy.2026.10240

**Published:** 2026-07-31

**Authors:** M. Puialto Amieiro, M. Tubio, E. Cernadas, M. Fernández Delgado, E. Real, M. D. P. Alonso, Á. Carracedo, M. Fernández Prieto, J. Segalas

**Affiliations:** 1 Hospital Álvaro Cunqueiro, Vigo; 2 CIMUS, Universidade de Santiago de Compostela; 3 CITIUS, Santiago de Compostela; 4 Hospital de Bellvitge, Barcelona; 5 USC, Universidade de Santiago de Compostela, Spain

## Abstract

**Introduction:**

Obsessive–compulsive disorder (OCD) carries a markedly higher risk of suicidal thoughts and behaviors than the general population. Risk is amplified by affective and anxiety comorbidities, childhood trauma, persistent psychological distress, and certain symptom dimensions. Despite these insights, no reliable strategy exists to predict suicide risk in OCD. Machine-learning approaches are emerging as powerful tools for risk prediction in psychiatry and have shown promise in OCD populations.

**Objectives:**

To develop and validate a **machine-learning predictive model** of suicide risk in OCD using routinely collected clinical and sociodemographic variables.

**Methods:**

Longitudinal study of **199 OCD patients** (mean follow-up 17.8 years). Input variables: sociodemographic data (age at onset/diagnosis, years untreated, sex), psychiatric comorbidities, substance use (alcohol, cannabis, stimulants, benzodiazepines, opioids; tobacco excluded), family history of OCD and suicide, medical comorbidities, childhood trauma (Childhood Trauma Questionnaire subscales), and OCD severity (Yale–Brown Obsessive Compulsive Scale).

Target variable: suicide risk classified into **three categories**—(1) no suicidal behavior, (2) suicidal ideation or thoughts of death, (3) suicide attempt or completed suicide.

Suicidal behaviors were identified through clinical interviews and confirmed in psychiatric records. Supervised *machine-learning* models (*linear discriminant analysis*, *naive Bayes*) were trained with leave-one-out cross-validation. Performance was assessed with **Cohen’s kappa, sensitivity, specificity, F1-score, and AUC**.

**Results:**

The optimal model identified **three key predictors: family history of suicide, affective comorbidity, and substance use (excluding tobacco)**. This combination achieved **κ = 26.8 %, sensitivity 71.4 %, specificity 74.4 %, F1 = 62.7 %, and AUC = 0.80**, indicating moderate predictive capability. Neither OCD severity (Y-BOCS) nor childhood trauma improved accuracy. In the cohort, **25 %** experienced suicidal ideation or behavior and **2.5 % (5 patients)** died by suicide.

**Image 1:**
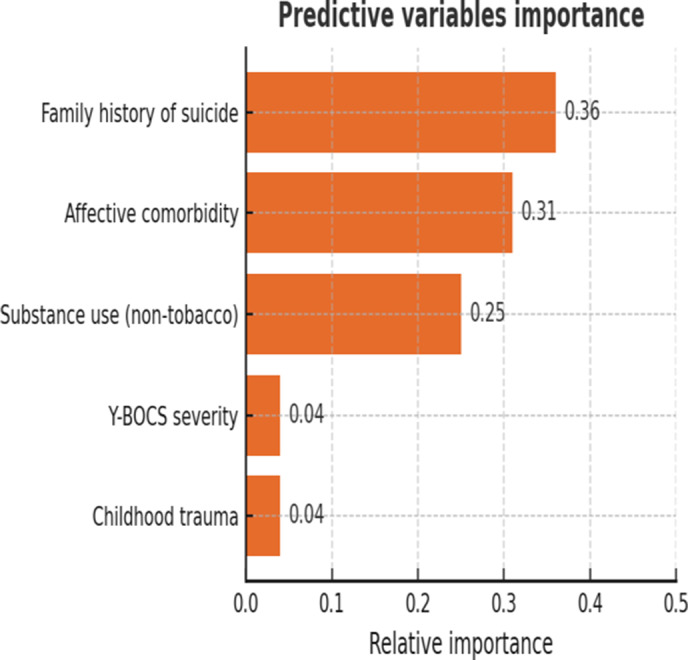

**Conclusions:**

A straightforward machine-learning model based on **standard clinical data** predicts suicide risk in OCD with moderate accuracy. The three predictors are routinely assessed, showing that **data-driven modeling validates traditional evaluation** for early detection and preventive monitoring.

**Clinical Take-Home Message:**

Machine-learning analysis **confirms and strengthens standard psychiatric assessment**. The presence of **family history of suicide, affective comorbidity, and substance use** provides sufficient information for a moderate-accuracy estimate of suicide risk in OCD, proving that **careful routine evaluation remains the cornerstone of early detection and targeted prevention**.

**Disclosure of Interest:**

None Declared

